# The Validation of Mortality Risk Indexes for Predicting Long-Term Outcomes in People Living with HIV in a Spanish Cohort (eVIHa)

**DOI:** 10.3390/jcm14248654

**Published:** 2025-12-06

**Authors:** Sophia Pinecki Socias, Marc Moragues Serra, Francisca Artigues Serra, Maria Luisa Martin, Javier Murillas, Aroa Villoslada, Adrian Rodriguez, Adelaida Rey, Julia Serra, Laia Vilaplana, Pedro Fernandez, Francisco Fanjul, Aina Millan, Melchor Riera Jaume

**Affiliations:** 1 Health Research Institute of the Balearic Islands (IdISBa), 07120 Palma de Mallorca, Spain; 2 Hospital Universitrio Son Espases, 07120 Palma de Mallorca, Spain; 3 Hospital Universitario Son Llàtzer, 07198 Palma de Mallorca, Spain; 4 Hospital Comarcar de Inca, 07300 Inca, Spain; 5 Hospital de Manacor, 07500 Manacor, Spain; 6 Hospital de Can Misses, 07800 Ibiza, Spain; 7 Faculty of Medicine, University of the Balearic Islands, 07122 Palma de Mallorca, Spain

**Keywords:** HIV, mortality, VACS index, Charlson index

## Abstract

**Background/Objectives**: Having access to antiretroviral therapy (ART) has altered the health status of people living with HIV (PLHIV) to that of having a chronic condition, with a greater life expectancy. The development of the Veterans Aging Cohort Study (VACS) Index has allowed for the prediction of 5-year mortality in PLHIV, using both HIV-related and non-HIV-related markers. The modified Charlson Index describes the comorbidity burden and is indicated to predict 10-year mortality. This study validates the Veterans Aging Cohort Study (VACS) Index 1.0 and the modified Charlson Index in a contemporary European cohort, with the aim of better predicting mortality. **Methods**: An observational, multicenter study was conducted using data from the eVIHa cohort in the Balearic Islands (Spain) from 2000 to 2023. The VACS Index 1.0 and the modified Charlson Index were calculated. Model discrimination was assessed using Harrell’s C-statistic, and observed mortality was estimated using Kaplan–Meier analysis. **Results**: Of 6913 eligible PLHIV, 4480 (64.8%) had sufficient data for VACS Index calculation and were included in the primary analysis. The excluded group (N = 2433) had significantly higher mortality (27.7% vs. 9.4%) and a greater proportion of people who inject drugs. In the analyzed cohort, the VACS Index 1.0 showed good discrimination for 5-year all-cause mortality (C-statistic: 0.759), outperforming the modified Charlson Index (C-statistic: 0.729). Discrimination was the highest for deaths from liver disease (C: 0.875) and non-HIV-related infections (C: 0.853). **Conclusions**: In our analyzed cohort, the VACS Index 1.0 accurately predicted 5-year mortality. However, its performance in populations with higher rates of people who inject drugs and irregular follow-up is unknown and likely to be lower. Clinicians should be aware of these limitations when applying the index in practice.

## 1. Introduction

Current antiretroviral treatments have enabled the control of Human Immunodeficiency Virus (HIV) infection, suppressing viral load with drugs that are easy to administer and have few adverse effects. Since the introduction of highly active antiretroviral therapy, mortality among people living with HIV (PLHIV) has decreased, mainly due to a reduction in Acquired Immunodeficiency Syndrome (AIDS)-related mortality. However, a lower life expectancy persists among PLHIV in developed countries due to an increase in non-AIDS-related deaths, especially from non-related malignancies, and cardiovascular or respiratory events [1,2,3,4].

Life expectancy is no longer viral-dependent and is more related to organ damages and associated comorbidities. Therefore, specific risk indexes have been developed over the years that integrate biomarkers of organ function and comorbidity indexes. Two of the most used ones are the Veterans Aging Cohort Study (VACS) Index and the Charlson Comorbidity Index (CCI). Both have been applied in adult cohorts and offer mortality risk predictions but differ in their included variables and construction [5]. The VACS Index was developed with health data from the VACS cohort and is based on routinely obtained HIV markers and non-HIV biomarkers of organ injury (Appendix A) [6]. It has demonstrated excellent 5-year mortality predictions, as seen in the NA-ACCORD collaboration [7], and has been validated in different cohorts in America and Europe. The VACS Index has been improved with the addition of albumin, white blood cell count (WBC), and body mass index (BMI) in VACS 2.0 [7,8]. While VACS 2.0 offers enhanced discrimination, its components may not be universally available in all clinical databases, especially in historical data. Therefore, the simpler VACS 1.0 index could have broader applicability if its predictive value remains robust in contemporary cohorts. The Charlson Comorbidity Index (CCI) is an older instrument and was developed for the general population. It weights different diseases and comorbidities based on diagnostic codes in order to estimate long-term mortality, including 10-year mortality. In PLHIV it has been used to characterize the global comorbidity burden. Since the CCI does not consider HIV markers nor the immunological status, its predictive power is more limited. The aim of this study is, therefore, to evaluate the utility and discrimination of VACS 1.0 and a modified CCI for predicting all-cause and cause-specific 5- and 10-year mortality in a large, population-based cohort of PLHIV in the modern era of antiretroviral therapy (ART).

## 2. Materials and Methods

### 2.1. Study Population

The cohort of PLHIV in the Balearic Islands (Spain) has been monitored since 1998 through the eVIHa clinical platform. The platform incorporates the participation of the four hospitals in Mallorca, along with hospitals in Menorca and Ibiza, which together provide nearly complete coverage for the care of PLHIV in this region. The cohort is open, multicenter, and observational, and included all newly diagnosed PLHIV or those transferred from another region or country, under medical follow-up at one of the hospitals in the Balearic Islands. Our study included all PLHIV from the cohort aged 18 years or older, who entered the cohort from 1 January 1985 to 31 December 2022, and were still in follow-up between 1 January 2000 and 31 December 2023, with at least two visits. The PLIV with insufficient data to calculate the VACS Index were excluded. This also meant that PLHIV with insufficient data to calculate the VACS index were excluded. All PLHIV provided informed consent, and the study was approved by the ethics committee of the Balearic Islands (IB3808.18).

### 2.2. Mortality Data

PLHIV were followed until the end of the study period, death, or loss to follow-up, and were censored at 5 and 10 years for survival analysis. Mortality data and causes were obtained from reviewed clinical records and cross-referenced with data from the National Institute of Statistics (INE).

### 2.3. Study Variables

The primary outcome was 5-year mortality from the baseline visit. The baseline visit was defined as the first clinical visit where all components for the VACS Index 1.0 calculation were available. Variables included sociodemographic data, HIV-associated variables, comorbidities, and the VACS 1.0 components (age, CD4 cell count, viral load, hemoglobin, FIB-4 Index, eGFR, and Hepatitis C coinfection) [6]. Although white blood cell counts were available, systematic measurements of albumin and WBC were not routinely collected in all participating hospitals before 2015, which prevented the calculation of VACS 2.0 for the majority of the cohort.

A modified Charlson Index was calculated. The original CCI assigns 6 points to AIDS, a weighting based on the prognosis of the pre-ART era [9]. In line with other contemporary analyses of HIV cohorts and to avoid overweighting this single condition in an era of effective viral suppression, we used a modified CCI where AIDS was assigned 1 point [10,11], as it showed better survival predictions.

### 2.4. Statistical Analysis

Baseline characteristics were analyzed descriptively. Categorical variables were expressed as numbers and percentages (compared with chi-square), and continuous variables as median and interquartile range (IQR) (compared with the Mann–Whitney U test). Observed mortality was estimated using Kaplan–Meier. Hazard ratios (HR) and 95% CIs for mortality were calculated using Cox models. Discrimination of VACS Index per 10 unit increase and the Charlson Index in predicting cause-specific mortality were assessed using Harrell’s C-statistic, which measures the ability of a predictive model to distinguish between individuals who experience an event earlier versus later (or not at all) using their predicted risk scores. C-statistic varies from 0.5 (no discrimination) to 1.0 (perfect discrimination). Analyses were additionally conducted for ten-year mortality to examine the potential of the indexes to predict mortality over time.

Statistical analysis was performed using SPSS 24.0. Significance level was set at <0.05.

## 3. Results

### 3.1. Study Population and Selection Bias

Of 7028 PLHIV in the cohort, 6913 met the inclusion criteria who had a total follow-up time of 79,713 person-years. Of these, 4480 (64.8%) had available data to calculate the VACS Index (analyzed group) and 2433 (35.2%) were excluded due to lack of data (non-analyzed group). The five-year follow-up time for the analyzed group was 19,086.51 person-years, with 206 deaths, resulting in a mortality rate of 10.79/1000 person-years. At 10 years, the rate was 11.98/1000 person-years.

The demographic characteristics of the analyzed cohort are shown in Table 1. The population consisted of 77.4% men, with a median age at diagnosis of 32 years. Advanced HIV disease was present in 28.6% of PLHIV (N = 1282), with a median CD4 nadir of 265 cells/µL (IQR: 120–425). Comorbidities were absent in only 32.6% of individuals. The most frequently reported comorbidities were hepatic disease (23.8%; N = 1068), followed by pulmonary disease (12.8%; N = 574) and diabetes (10.1%; N = 452). The median time from the first antiretroviral therapy (ART) prescription to the first available VACS assessment was 39.2 months (IQR: 9.0–138.9).

The comparison between the analyzed and non-analyzed groups (Table 2) revealed critical differences. The non-analyzed group had a significantly higher proportion of people who inject drugs (IDU) (29.2% vs. 22.7%) and of unknown transmission route (31.3% vs. 4.6%), irregular follow-up (73.7% vs. 20.6%), and a markedly higher cumulative mortality (27.7% vs. 9.4%, *p* < 0.001), especially of unspecified causes.

### 3.2. Index Validation

A survival analysis was conducted using Kaplan–Meier, comparing VACS index scores grouped between 0 and 35, 36 and 50, 51 and 70, and 71 and above, similarly to Bebu et al. [12]. The same survival analysis was performed for the modified Charlson Index grouped in 0–1, 2, and 3 or more points [5].

**Figure 1:**
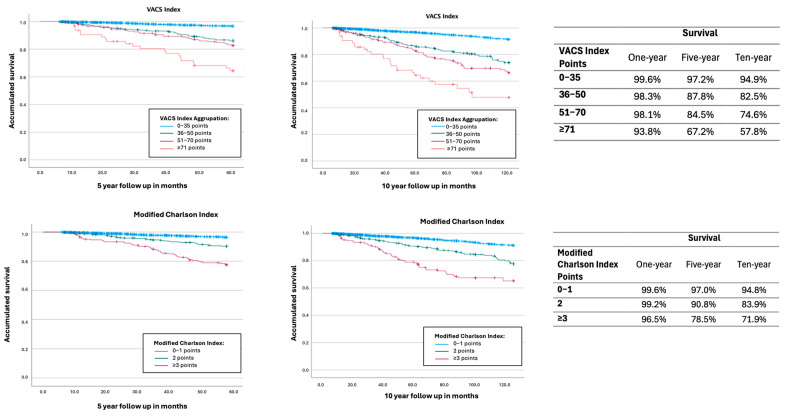
Kaplan–Meier curves for all-cause mortality for VACS Index aggrupation and modified Charlson Index aggrupation. VACS Index: Blue: 0–35 points; green: 36–50 points; pink: 51–70 points; red: 71 or more. Modified Charlson Index: Blue: 0–1 points; Green: 2 points; pink: 3 or more points.

In the analyzed cohort, the VACS Index score was strongly associated with being a good mortality predictor. The proportion of PLHIV who died within five years was 12.8% (110/861) in the group with VACS > 30, compared to only 0.8% (13/1609) in the 0–10 group (Table 1). Kaplan–Meier survival curves (Figure 1) showed significant differences between VACS risk strata (*p* < 0.001).

Causes of death were classified utilizing the Canadian classification. The main cause of death in five years (not considering unknown cause) was cancer (18.9% five-years and 20.4% ten-years) followed by infections other than HIV (15.5% five years and 14.8% ten years) (Table 3).

The Cox regression analysis (Table 3) showed that the VACS Index (per 10-unit increase) was significantly associated with a higher risk of all-cause death at 5 years (HR = 1.44, 95% CI: 1.38–1.51). The discrimination of the VACS Index (C-statistic = 0.759) was superior to that of the modified Charlson Index (C = 0.729). VACS discrimination was best for mortality due to liver disease (C = 0.875), non-HIV infections (C = 0.853), and HIV-related disease (C= 0.845).

Subgroup analyses showed that PLHIV over 50 years had a greater burden of comorbidities and a higher median VACS score, but the predictive power of the index was not superior in this group (Table 4 and Table 5), except for HIV-related causes of death, hepatic causes, and non-HIV infections.

The discrimination ability of the index was higher in the 2016–2023 period compared to 2000–2015 (Table 6).

Table 7 shows predicted versus observed mortality, indicating reasonable calibration.

## 4. Discussion

In this study of a large European population-based cohort, the VACS Index 1.0 proved to be a robust predictor of 5-year mortality, with discrimination superior to that of a modified Charlson Index. We observed a C-statistic of 0.759 for 5-year all-cause mortality, a value comparable to the original validation studies [6]. However, this result must be interpreted with caution, as it was derived from a cohort that excluded over one-third (35.2%) of the total cohort population.

The finding that the excluded group was characterized by a higher baseline mortality, greater injection drug use, and irregular follow-up is critically important. The VACS Index is constructed from markers that require regular clinical engagement. Therefore, the study design has an inherent bias that selects for a more stable and likely healthier population, which probably results in an optimistic estimation of the index’s performance. The true predictive ability of the VACS in a real-world, unselected population is therefore likely lower than what we report. This finding itself highlights that prognostic models based on clinical data may systematically fail to capture risk in the most vulnerable population.

Our cohort represents a population-based sample, including all individuals receiving HIV care in the Balearic Islands. This confers several strengths, such as demographic diversity—including women and immigrants—and access to high-quality clinical data. The integration of electronic health records and centralized laboratory systems ensures robust documentation of comorbidities and substance use. Furthermore, the cohort benefits from extended follow-up, minimal losses, and adherence to quality control standards consistent with national and international guidelines. Deaths and losses to follow-up were cross-validated through national and regional sources, including the National Institute of Statistics (INE) and the Balearic Health Department, allowing for reliable classification of causes of death. Importantly, unlike other studies, no imputation methods were used; PLHIV lacking the laboratory data necessary for calculating the VACS Index were excluded from the primary analyses.

Overall mortality in PLHIV for whom VACS Index 1.0 could be calculated was 10.79 deaths per 1000 person-years, notably lower than that reported in the original VACS, and more closely resembled figures from the ART-CC cohort [6,8]. This discrepancy is partly explained by the recency of our data, which includes individuals initiating ART after 2015, an era marked by universal treatment guidelines and widespread use of integrase strand transfer inhibitors. For individuals starting ART during this period, the ART-CC cohort reported a mortality rate of 8.2 per 1000 person-years [13]. The median VACS Index in our cohort was 16 points, mirroring that observed in the ART-CC validation study [6].

VACS 1.0 demonstrated strong predictive performance for five-year mortality in our cohort, with clear stratification of survival curves by VACS score categories. The hazard ratio (HR) for five-year mortality was 1.44 (95% CI: 1.38–1.51), which is higher than the HRs reported in the original VACS [1.22 (1.21–1.249)] and the ART-CC validation cohort [1.32 (1.27–1.38)], although the overall discriminatory power (C-statistic) was similar or slightly lower. Discrimination was highest for HIV-related deaths, chronic liver disease, and non-HIV infections, consistent with prior studies [6,13]. Although the median VACS Index was significantly higher in PLHIV aged over 50 (median 24), and mortality was accordingly greater (7.9%), predictive accuracy in this age group was not superior—an observation also noted in the VACS and ART-CC cohorts [6,8]. The discriminative capacity of the VACS 1.0 Index improved over time, being notably higher between 2016 and 2023 (C-statistic: 0.791) compared to the earlier period (C-statistic: 0.696), particularly for HIV- and liver-related causes of death.

This finding may be attributed to limitations in the calibration and discrimination of the VACS Index in certain subpopulations, such as individuals with CD4 counts below 200 cells/μL and women—groups that have historically been underrepresented in validation studies, which have largely focused on predominantly male veteran cohorts [7,8]. Alternatively, this improvement may reflect enhancements in the completeness and accuracy of laboratory data collection in more recent years, both in our cohort and in others.

Although the VACS Index effectively reflects an individual’s current health status, it does not account for longitudinal changes or cumulative exposures to risk factors, which may be important for long-term mortality prediction. This limitation could explain the stronger index performance in predicting short-term rather than long-term mortality outcomes [14].

Recent validation studies have demonstrated that the VACS Index 2.0 maintains robust discriminatory ability and calibration for predicting all-cause mortality in contemporary cohorts, particularly when applied to data from 2010 to 2018—a period marked by declining mortality rates and evolving clinical characteristics among PLHIV [7,13]. The incorporation of additional laboratory markers in the VACS Index 2.0 enhances its capacity to capture the effects of both HIV-related and non-HIV-related morbidity. This enhancement is increasingly pertinent as the aging PLHIV population faces a greater burden of comorbidities, which have become key determinants of mortality risk. Consequently, VACS 2.0 offers more accurate risk stratification and mortality probability estimates in recent years compared to older models that do not account for the emerging factors.

The predictive performance of VACS 1.0 diminished when assessing ten-year mortality. VACS 2.0, which incorporates additional variables such as albumin, white blood cell count, and body mass index, has demonstrated improved discrimination over longer follow-up periods, increasing the C-statistic from 0.776 to 0.805 in the VACS cohort and from 0.800 to 0.831 in ART-CC [8]. VACS 2.0 also enhances mortality prediction for certain causes of death, including chronic pulmonary disease, and has shown improved performance particularly among PLHIV with suppressed HIV-1 RNA levels [7].

The Charlson Comorbidity Index (CCI) is a widely used tool for estimating ten-year survival, incorporating patient age and comorbidities [9]. In our cohort, the prevalence of conditions such as renal failure and cancer was similar to that observed in the original VACS population. However, we observed a lower prevalence of diabetes mellitus and a higher prevalence of chronic liver disease (23.8%) and chronic obstructive pulmonary disease (COPD, 12.8%) [5]. In the study by McGinnis et al. [5], comparing comorbidities and the CCI between HIV-positive and HIV-negative populations, only chronic liver disease was more common in those living with HIV. Notably, despite a comparable comorbidity burden, the overall Charlson score was higher among people living with HIV, largely due to the heavy weighting of AIDS in the original index (6 points). In our study, we used a modified Charlson Index adjusted the AIDS weight to 1, aligning it with other comorbidities, as supported by previous findings [10], and in recognition of the reduced predictive accuracy observed when using the original scoring. McGinnis et al. also demonstrated that VACS 2.0 and a combined VACS-CCI provided superior prediction of ten-year mortality, although the latter has not been widely validated [5]. In the general and HIV-positive populations, the VACS-CCI showed excellent discrimination (C-statistic = 0.81), and when applied to people living with HIV, it yielded a similar predictive performance to VACS 2.0 (C = 0.77), closely aligning with our findings (C = 0.76).

Observed and predicted deaths were generally well aligned across specific causes, with deviations primarily occurring in individuals with VACS scores of 41–50 (higher than predicted mortality) and those with scores ≥ 81 (lower observed mortality).

### Limitations

The primary limitation of this study is the selection bias already discussed. A second limitation is the use of VACS 1.0 instead of VACS 2.0. We have framed this as a pragmatic choice; the lack of complete laboratory data (specifically albumin and WBC) for the historical cohort prevented the calculation of VACS 2.0. However, this reflects a real-world scenario in many clinical settings where only the components of VACS 1.0 are available. Thus, our study provides a valuable validation of the simpler index’s utility in a contemporary.

## 5. Conclusions

The VACS 1.0 is a valid predictor of 5-year mortality in people with HIV who receive regular clinical care. Its performance in more marginalized populations with irregular follow-up is unknown and likely to be lower. Clinicians should be aware of these limitations when applying the index in practice, and further research is needed to develop risk models that better capture vulnerability across all PLHIV subgroups. Even though VACS Index 1.0 had better mortality discrimination, the modified Charlon Index is also a good mortality predictor in our cohort. Our results provided great discrimination for VACS Index 1.0, but further studies should be performed with VACS Index 2.0 to provide better mortality risk estimates.

## Figures and Tables

**Table 1:** Demographic and clinical characteristics of the analyzed study population (N = 4480) by 5 years mortality.

Characteristics		Total (N = 4480)	No Mortality (N = 4274)	Mortality (N = 206)
Age cohort entry (years)	Median (IQR)	36 (30–43)	36 (30–43)	40 (32–51)
Age at infection (years)	Median (IQR)	32 (26–39)	32 (26–39)	34 (26–46)
Gender	N, (%)			
Male		3466 (77.4)	3301 (77.2)	165 (80.1)
Female		1014 (22.6)	973 (22.8)	41 (19.9)
Origin	N, (%)			
Europe		3435 (76.7)	3236 (75.7)	199 (96.6)
Africa		204 (4.6)	202 (4.7)	2 (1)
South America		812 (18.1)	808 (18.9)	4 (1.9)
Other		29 (0.6)	28 (0.7)	1 (0.5)
Transmission route	N, (%)			
Heterosexual		1394 (31.1)	1345 (31.5)	49 (23.8)
MSM		1804 (40.3)	1771 (41.4)	33 (16)
IDU		1017 (22.7)	909 (21.3)	108 (52.4)
Other		61 (1.4)	59 (1.4)	2 (1)
Unknown		204 (4.6)	190 (4.4)	14 (6.8)
Regular follow-up	N, (%)	3556 (79.4)	3400 (79.6)	156 (75.7)
CD4 nadir (cells/μL)	Median (IQR)	265 (120–425)	272 (127–433)	110 (44–256)
CD4 at start of ART (cells/μL) (N = 2689)	Median (IQR)	314 (163–486)	319 (170–491) (N = 2588)	184 (83–315) (N = 101)
Last CDC stage	N, (%)			
A		2762 (61.8)	2685 (63)	77 (37.6)
B		613 (13.7)	579 (13.6)	34 (16.6)
C		1089 (24.4)	995 (23.3)	94 (45.9)
Advanced HIV	N, (%)	1282 (28.6)	1175 (27.5)	
Tobacco use	N, (%)			
Non smoker		1886 (42.1)	1782 (41.7)	104 (50.5)
Ever smoker		2594 (57.9)	2492 (58.3)	102 (49.5)
Packages/year (N = 2083)	Median (IQR)	24 (11.2–39)	23.4 (11–38.7) (N = 1998)	36.7 (18.7–47) (N = 85)
N° comorbidities	N, (%)			
0		1461 (32.6)	1432 (33.5)	29 (14.1)
1		1177 (26.3)	1105 (25.9)	72 (35)
2		762 (17)	727 (17)	35 (17)
≥3		1080 (24.1)	1010 (23.6)	70 (34)
Comorbidities				
Diabetes	N, (%)	452 (10.1)	424 (9.9)	28 (13.6)
Cancer	N, (%)	304 (6.8)	255 (6)	49 (23.8)
Hematological cancer	N, (%)	46 (1)	40 (0.9)	6 (2.9)
Cardiac insufficiency	N, (%)	103 (2.3)	93 (2.2)	10 (4.9)
Hepatic disease	N, (%)	1068 (23.8)	958 (22.4)	110 (53.4)
Pulmonary disease	N, (%)	574 (12.8)	541 (12.7)	33 (16)
Renal disease	N, (%)	355 (7.9)	329 (7.7)	26 (12.6)
VACS Index	Median (IQR)	16 (6–27)	15 (6–25)	33 (21–52)
VACS Index	N, (%)			
0–10		1609 (35.9)	1596 (37.3)	13 (6.3)
11–20		1231 (27.5)	1195 (28)	36 (17.5)
21–30		779 (17.4)	732 (17.1)	47 (22.8)
>30		861 (19.2)	751 (17.6)	110 (53.4)
Modified Charlson Index	N, (%)			
0–1		3373 (84.2)	3360 (85.6)	113 (54.9)
2		479 (10.7)	435 (10.2)	44 (21.4)
≥3		228 (5.1)	179 (4.2)	49 (23.8)
Time between HIV diagnosis and ART start (months)	Median (IQR)	20 (2–81) (N = 4424)	19 (2–80) (N = 4233)	24 (4–106) (N = 191)
Time between ART and first available VACS (months)	Median (IQR)	39.2 (9–138.9) (N = 4042)	35.3 (8.8–131.8) (N = 4274)	120 (40–206.9) (N = 206)
Time follow-up from first VACS for 5 years (months)	Median (IQR)	59.97 (48.0–60.0)	59.97 (53.5–60.0)	30.95 (18.83–44.37)
Time between cohort entry and first available VACS (months)	Median (IQR)	20.49 (7.59–125.92)	18.67 (7.37–120.1)	99.75 (19.57–181.07)
N° visit post 1998	Median (IQR)	28 (15–48)	29 (15–48)	21 (10–48)

Abbreviations: IQR: Interquartile range; MSM: Men who have sex with men; IDU: People who inject drugs.

**Table 2:** Comparison of characteristics between analyzed and non-analyzed PLHIV.

Characteristic		Non-Analyzed Group (N = 2433)	Analyzed Group (N = 4480)	*p*-Value
Age at cohort entry (years)	Median (IQR)	37 (31–45)	36 (30–43)	<0.001
Age at infection (years)	Median (IQR)	33 (27–42)	32 (26–39)	<0.001
Gender	N, (%)			
Male		1905 (78.3)	3466 (77.4)	0.374
Female		528 (21.7)	1014 (22.6)	
Origin	N, (%)			
Europa		1979 (81.6)	3435 (76.7)	<0.001
Africa		88 (3.6)	204 (4.6)	
South America		348 (14.3)	812 (18.1)	
Other		11 (0.5)	29 (0.6)	
Transmission route	N, (%)			
Heterosexual		434 (17.9)	1394 (31.1)	<0.001
MSM		504 (20.7)	1804 (40.3)	
IDU		709 (29.2)	1017 (22.7)	
Other		21 (0.9)	61 (1.4)	
Unknown		761 (31.3)	204 (4.6)	
CD4 nadir (cells/μL)	Median (IQR)	254 (106–496) (N = 2022)	265 (120–425)	0.372
CD4 at start of ART (cells/μL)	Median (IQR)	253 (108–468) (N = 949)	314 (163–486) (N = 2689)	<0.001
Months between diagnosis and ART start	Median (IQR)	42 (4–101) (N = 2154)	20 (2–81) (N = 4424)	<0.001
Last CDC stage	N, (%)			
A		1375 (56.7)	2762 (61.8)	<0.001
B		372 (15.4)	613 (13.7)	
C		675 (27.9)	1089 (24.4)	
Tobacco use	N, (%)			
Non smoker		1785 (73.4)	1886 (42.1)	<0.001
Ever smoker		648 (26.6)	2594 (57.9)	
N° comorbidities	N, (%)			
0		1428 (58.7)	1461 (32.6)	<0.001
1		586 (24.1)	1177 (26.3)	
2		209 (8.6)	762 (17)	
≥3		210 (8.6)	1080 (24.1)	
Comorbidities				
Diabetes	N, (%)	74 (3)	452 (10.1)	<0.001
Cancer	N, (%)	132 (5.4)	304 (6.8)	0.026
Hematological cancer	N, (%)	21 (0.9)	46 (1)	0.507
Cardiac Insufficiency	N, (%)	29 (1.2)	103 (2.3)	<0.001
Hepatic disease	N, (%)	660 (27.1)	1068 (23.8)	0.003
Pulmonary disease	N, (%)	101 (4.2)	574 (12.8)	<0.001
Renal disease	N, (%)	61 (2.5)	355 (7.9)	<0.001
Regular follow-up	N, (%)	640 (26.3)	3556 (79.4)	<0.001
Modified Charlson Index at end of follow-up	N, (%)			
0–1		1996 (83.2)	3325 (74.2)	<0.001
2		269 (11.2)	605 (13.5)	
≥3		134 (5.6)	550 (12.3)	
N° visits post 1998	Median (IQR)	77 (28–149)	147 (73–242)	<0.001
Months between HIV infection and exitus	Median (IQR)	153 (91–218) (N = 672)	230 (140–295) (N = 421)	
Total Mortality	N, (%)	673 (27.7)	421 (9.4)	<0.001
Death cause	N, (%)			
HIV-related		69 (10.3)	23 (5.5)	<0.001
Accidents		14 (2.1)	7 (1.7)	
Cancer		100 (14.9)	90 (21.4)	
Not specified cause		266 (39.6)	124 (29.5)	
Hepatic		73 (10.9)	27 (6.4)	
Cardiovascular		22 (3.3)	30 (7.1)	
Infections not related to HIV		83 (12.4)	57 (13.5)	
Neurological		11 (1.6)	10 (2.4)	
Renal		2 (0.3)	1 (0.2)	
Pulmonary		2 (0.3)	9 (2.1)	
Drug abuse		24 (3.6)	31 (7.4)	
Suicide		6 (0.9)	12 (2.9)	
Age at end of follow-up (years)	Median (IQR)	44 (37–54)	51 (42–58)	<0.001
Months of follow-up	Median (IQR)	77 (28–149)	147 (73–242)	<0.001

*p*-value reflect comparison between the two groups. Abbreviations: IQR: interquartile range; MSM: Men who have sex with men; IDU: People who injects drugs.

**Table 3:** Hazard ratio (HR) and C-statistic (C) for the different causes of death for 5- and 10- year mortality for VACS Index and modified Charlson Index.

Period	Cause of Death	Deaths (N)	VACS Index (per 10 u) HR (95% IC)	C-Statistic	Modified Charlson Index HR (95% IC)	C-Statistic
5-year mortality	All-cause death	206	1.44 (1.38–1.51)	0.759	1.73 (1.60–1.86)	0.729
	HIV-related	16	1.55 (1.31–1.77)	0.845	1.64 (1.23–2.18)	0.719
	Accidental/Suicide/substance abuse	28	1.31 (1.13–1.51)	0.666	1.38 (1.06–1.80)	0.639
	Cancer	39	1.38 (1.23–1.54)	0.757	1.79 (1.52–2.11)	0.698
	Hepatic	12	1.68 (1.44–1.95)	0.875	2.05 (1.59–2.65)	0.813
	Cardiovascular	16	1.45 (1.24–1.71)	0.771	1.80 (1.4–2.32)	0.737
	Non-HIV infection	32	1.61 (1.46–1.77)	0.853	1.9 (1.61–2.25)	0.808
	Other causes	63	1.35 (1.23–1.48)	0.707	1.66 (1.44–1.91)	0.732
10-year mortality	All-cause death	339	1.40 (1.35–1.46)	0.742	1.69 (1.59–1.8)	0.711
	HIV-related	22	1.46 (1.27–1.67)	0.820	1.71 (1.35–2.17)	0.697
	Accidental/Suicide/substance abuse	44	1.23 (1.01–1.40)	0.637	1.4 (1.12–1.74)	0.644
	Cancer	69	1.37 (1.26–1.50)	0.746	1.62 (1.4–1.87)	0.657
	Hepatic	20	1.59 (1.41–1.80)	0.835	1.94 (1.56–2.41)	0.797
	Cardiovascular	22	1.49 (1.31–1.70)	0.781	1.81 (1.45–2.26)	0.736
	Non-HIV infection	50	1.53 (1.41–1.66)	0.819	1.86 (1.61–2.14)	0.784
	Other causes	111	1.34 (1.025–1.43)	0.701	1.67 (1.49–1.87)	0.720

Hazard ratio indicates the risk of mortality due to the indicated cause of death, indicating its confidence interval. C-statistic indicates level of discrimination for each index and cause of death. Abbreviation: Per 10 u: Per 10 units increase.

**Table 4:** Characteristics cohort under and over 50 years.

Characteristics		<50 Years (N = 3096)	≥50 Years (N = 1384)	*p*-Value
Age cohort entry (years)	Median (IQR)	34 (29–39)	47 (36–53)	<0.001
Age at infection (years)	Median (IQR)	30 (25–36)	40 (31–50)	<0.001
Age at end follow-up (max 5 years) (years)	Median (IQR)	46 (39–51)	60 (57–66)	<0.001
Gender	N, (%)			
Male		2407 (77.7)	1059 (76.5)	0.364
Female		689 (22.3)	325 (23.5)	
Origin	N, (%)			
Europe		2229 (72)	1206 (87.1)	<0.001
Africa		168 (5.4)	36 (2.6)	
South America		675 (21.8)	137 (9.9)	
Others		24 (0.8)	5 (0.4)	
Transmission route	N, (%)			
Heterosexual		908 (29.3)	486 (35.1)	<0.001
MSM		1375 (44.4)	429 (31)	
IDU		648 (20.9)	369 (26.7)	
Other		53 (1.7)	8 (0.6)	
Unknown		112 (3.6)	92 (6.6)	
Regular follow-up	N, (%)	2358 (76.2)	1198 (86.6)	<0.001
CD4 nadir (cells/μL))	Median (IQR)	272 (127–433)	110 (44–256)	<0.001
CD4 at start of ART (cells/μL) (N = 2689)	Median (IQR)	319 (170–491) (N = 2588)	184 (83–315) (N = 101)	<0.001
Last CDC stage	N, (%)			
A		2061 (66.7)	701 (50.7)	<0.001
B		358 (11.6)	255 (18.5)	
C		667 (21.6)	422 (30.5)	
Tobacco use	N, (%)			
Non smoker		1408 (45.5)	478 (34.5)	<0.001
Ever smoker		1688 (54.5)	906 (65.5)	
N° comorbidities	N, (%)			
0		1253 (40.5)	208 (15)	<0.001
1		894 (28.9)	283 (20.4)	
2		455 (14.7)	307 (22.2)	
≥3		494 (16)	586 (42.3)	
Comorbidities				
Diabetes	N, (%)	208 (6.7)	244 (17.6)	<0.001
Cancer	N, (%)	121 (3.9)	183 (13.2)	0.698
Hematological Cancer	N, (%)	33 (1.1)	13 (0.9)	<0.001
Cardiac insufficiency	N, (%)	29 (0.9)	74 (5.3)	<0.001
Hepatic disease	N, (%)	680 (22)	388 (28)	<0.001
Pulmonary disease	N, (%)	303 (9.8)	271 (19.6)	<0.001
Renal disease	N, (%)	131 (4.2)	224 (16.2)	<0.001
VACS Index	Median (IQR)	10 (5–20)	24 (18–37)	<0.001
VACS Index	N, (%)			
0–35		2827 (91.3)	1015 (73.3)	<0.001
36–50		160 (5.2)	201 (14.5)	
51–70		91 (2.9)	122 (8.8)	
71 or more		18 (0.6)	46 (3.3)	
Modified Charlson Index	N, (%)			
0–1		2759 (89.1)	1014 (73.3)	<0.001
2		258 (8.3)	221 (16)	
≥3		79 (2.6)	149 (10.8)	
Time between HIV diagnosis and ART start (months)	Median (IQR)	19 (3–73)	24 (3–100)	<0.001
Time between cohort entry and first available VACS (months)	Median (IQR)	12.8 (6.7–67.32)	125.57 (12.1–218.74)	<0.001
Time between first VACS and end of follow-up (5 years) (months)	Median (IQR)	59.97 (48–60)	59.97 (48–60)	0.221
N° visit post 1998	Median (IQR)	24 (14–39)	40 (31–50)	<0.001
Exitus in five years	N, (%)	96 (3.1)	110 (7.9)	<0.001

Abbreviations: MSM: Men who have sex with men; IDU: People who injects drugs.

**Table 5:** Hazard ratio (HR) and C-statistic (C) for 5-year mortality by age groups.

	<50 Years (N = 3096)					≥50 Years (N = 1384)				
	Deaths (N)	VACS Index Score	VACS Index per 10 Units Increase			Deaths (N)	VACS Index Score	VACS Index per 10 Units Increase		
Cause of Death	N	Median (IQR)	HR (95% CI)	*p*	C-Statistic	N	Median (IQR)	HR (95% CI)	*p*	C-Statistic
All cause death	96	23 (14–42)	1.503 (1.385–1.632)	<0.001	0.743	110	45 (24–61)	1.368 (1.284–1.457)	<0.001	0.724
HIV-related	12	32.5 (38–48.5)	1.665 (1.350–2.053)	<0.001	0.871	4	67 (51.5–79)	1.627 (1.251–2.117)	<0.001	0.934
Accidental/suicide/ substance abuse	17	18 (12–28)	1.397 (1.128–1.73)	0.002	0.667	11	39 (23–48)	1.252 (0.988–1.586)	0.062	0.679
Cancer	7	17 (15–22)	1.221 (0.821–1.814)	0.324	0.698	32	31 (22.5–53.5)	1.217 (1.052–1.407)	0.008	0.633
Hepatic	7	34 (19–103)	1.985 (1.547–2.546)	<0.001	0.880	5	62 (58–80)	1.595 (1.257–2.024)	<0.001	0.898
Cardiovascular	6	21 (6–42)	1.367 (0.948–1.97)	0.094	0.702	10	38.5 (32–66)	1.399 (1.145–1.71)	0.001	0.765
Non-HIV infection	8	38 (14–47)	1.655 (1.28–2.139)	<0.001	0.790	24	52 (36–72)	1.495 (1.330–1.681)	<0.001	0.816
Other causes	39	23 (11–37)	1.427 (1.246–1.635)	<0.001	0.712	24	39 (23–64.5)	1.322 (1.145–1.527)	<0.001	0.686

Hazard ratio indicates the risk of mortality due to the indicated cause of death, indicating its confidence interval. C-statistic indicates level of discrimination for each index and cause of death. *p*-value reflect comparison between the two groups, with significance level set at <0.05. Abbreviation: IQR: Interquartile range; IC: Confidence interval.

**Table 6:** Hazard ratio (HR) and C-statistic (C) for 5-year mortality by time period.

	2000–2015 (N = 1376)					2016–2023 (N = 3104)				
	Deaths	VACS Index Score	VACS Index per 10 Units Increase			Deaths	VACS Index Score	VACS Index per 10 Units Increase		
Cause of Death	N	Median (IQR)	HR (95% IC)	*p*-Value	C-Statistic	N	Median (IQR)	HR (95% IC)	*p*-Value	C-Statistic
All-cause death	97	34 (19–50)	1.33 (1.241–1.425)	<0.001	0.696	109	33 (23–55)	1.543 (1.499–1.643)	<0.001	0.791
HIV-related	12	32.5 (28–46.5)	1.335 (1.099–1.622)	0.004	0.757	4	55.5 (51–67)	1.849 (1.421–2.407)	<0.001	0.970
Accidental/suicide/substance abuse	6	23 (13–47)	1.137 (0.789–1.639)	0.491	0.561	22	23 (17–43)	1.41 (1.2–1.657)	<0.001	0.706
Cancer	13	29 (18–50)	1.278 (1.042–1.568)	0.019	0.684	26	27 (18–47)	1.464 (1.273–1.685)	<0.001	0.795
Hepatic	7	34 (19–103)	1.511 (1.224–1.865)	<0.001	0.772	5	62 (58–80)	1.911 (1.515–2.41)	<0.001	0.956
Cardiovascular	6	30 (21–50)	1.371 (1.055–1.782)	0.018	0.665	10	36 (23–43)	1.532 (1.243–1.888)	<0.001	0.823
Non-HIV infection	12	47 (25–79.5)	1.503 (1.28–1.764)	<0.001	0.766	20	51.5 (30.5–62.5)	1.72 (1.516–1.951)	<0.001	0.897
Other causes	41	33 (19–46)	1.261 (1.123–1.417)	<0.001	0.672	22	26 (12–52)	1.398 (1.186–1.648)	<0.001	0.690

Hazard ratio indicates the risk of mortality due to the indicated cause of death, indicating its confidence interval. C-statistic indicates level of discrimination for each index and cause of death. *p*-value reflect comparison between the two groups, with significance level set at <0.05. Abbreviation: IQR: Interquartile range; IC: Confidence interval.

**Table 7:** Predicted and observed mortality for all cause death and specific death cause for the VACS Index in 10 points increase.

		VACS Index								
	Mortality	0–10	11–20	21–30	31–40	41–50	51–60	61–70	71–80	≥81
All-cause death	Predicted	1.96%	3.11%	4.47%	6.52%	8.82%	13.12%	17.71%	24%	64.89%
	Observed	0.81%	2.92%	6.03%	6.51%	15.50%	12.77%	20.83%	32.35%	33.33%
HIV-related	Predicted	0.12%	0.21%	0.32%	0.49%	0.70%	1.11%	1.57%	2.23%	7.26%
	Observed	0.00%	0.16%	0.51%	0.26%	2.00%	1.42%	1.39%	2.94%	3.33%
Accidental/suicide/substance abuse	Predicted	0.35%	0.51%	0.66%	0.88%	1.07%	1.46%	1.78%	2.17%	4.33%
	Observed	0.12%	0.65%	0.90%	0.52%	2.50%	1.42%	0.00%	2.94%	3.33%
Cancer	Predicted	0.43%	0.65%	0.89%	1.24%	1.60%	2.27%	2.92%	3.76%	8.80%
	Observed	0.00%	0.89%	1.41%	1.04%	2.50%	3.55%	1.39%	5.88%	0.00%
Hepatic disease	Predicted	0.06%	0.11%	0.19%	0.33%	0.51%	0.88%	1.38%	2.09%	9.85%
	Observed	0.00%	0.16%	0.26%	0.26%	0.50%	0.71%	1.39%	2.94%	10.00%
Cardiovascular disease	Predicted	0.15%	0.24%	0.34%	0.51%	0.69%	1.03%	1.41%	1.91%	5.31%
	Observed	0.12%	0.00%	0.51%	1.04%	1.50%	0.00%	2.78%	0.00%	3.33%
Non-HIV infection	Predicted	0.20%	0.36%	0.57%	0.93%	1.40%	2.31%	3.51%	5.19%	20.52%
	Observed	0.06%	0.24%	0.51%	0.78%	3.00%	3.55%	2.78%	11.76%	13.33%
Other causes	Predicted	0.73%	1.08%	1.46%	1.99%	2.54%	3.53%	4.45%	5.67%	12.69%
	Observed	0.50%	0.81%	1.93%	2.60%	3.50%	2.13%	11.11%	5.88%	0.00%

## Data Availability

The raw data supporting the conclusions of this article will be made available by the authors on request.

## Appendix A

**Table A1:** Components from VACS Index 1.0 and the modified Charlson Index.

VACS 1.0		Points	Modified Charlson Index	Points
Age	<50 years	0	Myocardial infarction	1
	50–64 years	12	Congestive heart failure	1
	≥65 years	27	Peripheral vascular disease	1
CD4 count	≥500 cells/mm^3^	0	Cerebrovascular disease	1
	350–499 cells/mm^3^	6	Dementia	1
	200–349 cells/mm^3^	6	Chronic pulmonary disease	1
	100–199 cells/mm^3^	10	Connective tissue disease	1
	50–100 cells/mm^3^	28	Peptic ulcer disease	1
	<50 cells/mm^3^	29	Mild liver disease	1
HIV-1 RNA	<50 copies/mL	0	Diabetes without complications	1
	500–99,999 copies/mL	7	Hemiplegia	2
	≥1 × 10^5^ copies/mL	14	Moderate to severe renal disease	2
Hemoglobin	≥14 g/dL	0	Diabetes with end organ damage	2
	12–13.9 g/dL	10	Any tumor	2
	<10 g/dL	38	Leukemia	2
FIB-4 Index	<1.45	0	Lymphoma	2
	1.45–3.25	6	Moderate to severe liver disease	3
	>3.25	25	Metastatic solid tumor	6
eGFR	≥60 mL/min	0	AIDS	6
	45–59.9 mL/min	6		
	30–44.9 mL/min	8		
	<30 mL/min	26		
Hepatitis C co-infection	No	0		
	Yes	5		
